# Low-carbohydrate dietary pattern on glycemic outcomes trial (ADEPT) among individuals with elevated hemoglobin A1c: study protocol for a randomized controlled trial

**DOI:** 10.1186/s13063-020-05001-x

**Published:** 2021-02-01

**Authors:** Kirsten S. Dorans, Lydia A. Bazzano, Lu Qi, Hua He, Lawrence J. Appel, Jonathan M. Samet, Jing Chen, Katherine T. Mills, Bernadette T. Nguyen, Matthew J. O’Brien, Gabriel I. Uwaifo, Jiang He

**Affiliations:** 1 Department of Epidemiology, Tulane University School of Public Health and Tropical Medicine, 1440 Canal St, Suite 2000, New Orleans, LA 70112 USA; 2 Department of Medicine, Tulane University School of Medicine, New Orleans, LA USA; 3 Welch Center for Prevention, Epidemiology, and Clinical Research, Johns Hopkins University Bloomberg School of Public Health, Baltimore, MD USA; 4 Colorado School of Public Health, Aurora, CO USA; 5 Division of General Internal Medicine and Geriatrics, Department of Medicine, Institute of Public Health and Medicine, Chicago, IL USA; 6 Department of Preventive Medicine, Northwestern University Feinberg School of Medicine, Chicago, IL USA; 7 Department of Endocrinology, Diabetes, Metabolism, and Weight Management, Ochsner Medical Center, New Orleans, LA USA

**Keywords:** Prediabetes, Diabetes, Low-carbohydrate diet, Behavioral dietary intervention

## Abstract

**Background:**

Type 2 diabetes mellitus (T2DM) is a major cause of morbidity and mortality globally. Strong evidence supports the importance of diet and other lifestyle factors in preventing T2DM. Among individuals with T2DM, low-carbohydrate diets lead to decreases in hemoglobin A1c (HbA1c). However, research on the effects of low-carbohydrate diets on glycemic outcomes among individuals not currently on glucose-lowering medications who have elevated HbA1c is limited.

**Methods:**

The objective of this randomized controlled trial is to study the effect of a healthy low-carbohydrate diet achieved through behavioral intervention and key food supplementation compared with usual diet on HbA1c and other metabolic risk factors among individuals with HbA1c from 6.0 to 6.9% who are not on glucose-lowering medications. In this parallel trial, 150 participants will be randomized to the intervention or control group for 6 months. The healthy low-carbohydrate diet target is < 40 g of net carbohydrates during the first 3 months and < 40 to 60 net grams for months 3 to 6. This diet is characterized by abundant unsaturated fat and protein, high-fiber foods such as non-starchy vegetables and nuts, and minimal refined carbohydrates. The primary outcome is the difference in HbA1c change from baseline to 6 months in the intervention compared with usual diet group. Secondary outcomes include differences between groups in 6-month changes in fasting glucose, systolic blood pressure, total-to-high-density lipoprotein (HDL) cholesterol ratio, and body weight. Exploratory outcomes include differences in 6-month changes in fasting insulin, homeostasis model assessment of insulin resistance, diastolic blood pressure, waist circumference, and 10-year cardiovascular disease risk. An intention-to-treat analysis will be used.

**Discussion:**

We expect that the results from this study will lead to new approaches for developing and implementing dietary approaches (other than the most commonly used reduced fat diet) that will substantially reduce risk of cardiometabolic disease among adults with or at high risk of T2DM. The study intervention involves behavioral counseling and promotes consumption of dietary components thought to reduce risk of cardiometabolic disease and has expected applicability in clinical practice.

**Trial registration:**

ClinicalTrials.gov NCT03675360. Registered on September 18, 2018 (prior to enrolment of the first participant).

## Administrative information

The order of the items has been modified to group similar items (see http://www.equator-network.org/reporting-guidelines/spirit-2013-statement-defining-standard-protocol-items-for-clinical-trials/).

**Table:** 

Title {1}	Low-carbohydrate dietary pattern on glycemic outcomes trial (ADEPT) among individuals with elevated hemoglobin A1c: study protocol for a randomized controlled trial
Trial registration {2a and 2b}.	ClinicalTrials.gov registry: https://clinicaltrials.gov/ct2/show/NCT03675360. Registered on September 18, 2018.
Protocol version {3}	Version 2.2, Date: September 14, 2020
Funding {4}	This work was supported by the National Institute of General Medical Sciences (grant 1P20GM109036-01A1).
Author details {5a}	**Department of Epidemiology, Tulane University School of Public Health and Tropical Medicine, New Orleans, LA** Kirsten S. Dorans, Lydia A. Bazzano, Lu Qi, Hua He, Jing Chen, Katherine T. Mills, Bernadette T. Nguyen, Jiang He **Department of Medicine, Tulane University School of Medicine, New Orleans, LA** Lydia A. Bazzano, Jing Chen, Jiang He **Welch Center for Prevention, Epidemiology, and Clinical Research, Johns Hopkins University Bloomberg School of Public Health, Baltimore, MD** Lawrence J. Appel **Colorado School of Public Health, Aurora, Colorado** Jonathan M. Samet **Division of General Internal Medicine and Geriatrics, Department of Medicine; Institute of Public Health and Medicine; Department of Preventive Medicine, Northwestern University Feinberg School of Medicine, Chicago, IL, USA** Matthew J. O’Brien **Department of Endocrinology, Diabetes, Metabolism, and Weight Management, Ochsner Medical Center, New Orleans, Louisiana** Gabriel I. Uwaifo
Name and contact information for the trial sponsor {5b}	Kirsten S. Dorans, ScD Department of Epidemiology Tulane University School of Public Health and Tropical Medicine 1440 Canal St, Suite 2000, New Orleans, LA 70112 Phone: 504.988.0883 Email: kdorans@tulane.edu
Role of sponsor {5c}	The investigators are responsible for study design, collection, management, analysis, and interpretation of the data; writing of the report; and the decision to submit the report for publication.

## Introduction

### Background and rationale {6a}

As the 7th leading cause of mortality in the USA [1] and worldwide [2], diabetes is associated with a 2-fold higher risk of cardiovascular disease (CVD) [3] and can lead to other macrovascular and microvascular complications, including kidney damage, vision loss, peripheral arterial disease, and diabetic neuropathy [4–7]. Prediabetes, defined by the American Diabetes Association (ADA) as impaired fasting glucose (IFG), impaired glucose tolerance (IGT), or elevated hemoglobin A1c (HbA1c), is also referred to as “intermediate hyperglycemia” [8]. The US Centers for Disease Control (CDC) estimated that in 2015, 33.9% of US adults had prediabetes based on fasting glucose or HbA1c [9]. Approximately 5–10% of individuals with prediabetes develop diabetes within 1 year [10].

Globally, transitions towards reduced physical activity and increased refined carbohydrate intake have led to increased diabetes prevalence [11]. However, much evidence supports the role of healthy lifestyle change for preventing type 2 diabetes mellitus (T2DM) [12–16]. The Diabetes Prevention Program (DPP) study, which combined calorie restriction and exercise, reduced risk of T2DM by 58% among individuals with elevated fasting plasma glucose (FPG) and impaired glucose tolerance (IGT) [12]. The lifestyle intervention group had reduced risk of T2DM at 15 years [17]; similar benefits have been shown in trials internationally [18, 19].

The standard dietary approach to reduce risk of T2DM focuses on reducing caloric intake and total fat intake. However, increasing evidence suggests that types of fat, rather than quantity, may be more important. For example, a Mediterranean-style diet that does not restrict fat intake reduces risk of developing both T2DM and CVD [20, 21]. Moreover, low-to-moderate carbohydrate diets (< 45% energy from carbohydrates) are at least as effective as low-fat diets at promoting weight loss and improving CVD risk factors [22, 23]. Carbohydrates are the most important dietary determinant of postprandial blood glucose [24, 25]. In a meta-analysis of trials among individuals with T2DM, larger carbohydrate restriction corresponded with larger HbA1c reductions [26]. Some low-carbohydrate diets increase consumption of food items (e.g., red meat) associated with higher cardiometabolic disease risk [27]. However, foods high in carbohydrates can be replaced with foods common in Mediterranean-style diets (olive oil, nuts, non-starchy vegetables) [28] to create a healthy low-carbohydrate diet. In individuals with T2DM, low-carbohydrate Mediterranean diets led to greater improvement in glycemic control than higher carbohydrate diets [29–31].

Research focused on the glycemic effects of low-to-moderate carbohydrate diets among individuals with prediabetes is limited. In a non-randomized study, participants with IGT who participated in a 7-day in-hospital program (consisting of education and eating a low-carbohydrate diet) had reduced weight, HbA1c, and other glycemic outcomes at 12 months [32]. A small, single-arm pilot study of a low-carbohydrate DPP found a reduction in HbA1c at 6 months that was not sustained at 12 months [33]. A randomized pilot trial of overweight or obese individuals with T2D or prediabetes found that a very low-carbohydrate diet improved HbA1c and led to higher medication discontinuation and more weight loss compared with a low-fat diet [34, 35]. However, only four participants in this trial had prediabetes. Given benefits of low-to-moderate carbohydrate diets for weight loss in general populations and for glycemic control among patients with T2DM, further study of these diets is warranted among individuals with prediabetes and T2DM who are not currently on glucose-lowering medications.

### Objectives {7}

The goal of this randomized controlled trial is to study the effect of a healthy low-carbohydrate diet implemented through behavioral intervention (four weekly individual sessions, four biweekly group sessions, and three monthly group sessions) and key food supplementation compared with usual diet on metabolic risk factors among individuals with HbA1c 6.0–6.9% who are not taking glucose-lowering medications. The healthy low-carbohydrate diet target is < 40 g net carbohydrates (total carbohydrates minus fiber) for the first 3 months and < 40–60 net grams of carbohydrates for months 3 to 6. This diet is characterized by abundant unsaturated fats and protein, high-fiber foods such as non-starchy vegetables and nuts, and minimal refined carbohydrates.

Our *primary hypothesis* is that compared with usual diet, a healthy low-carbohydrate dietary pattern will lead to a larger decrease of HbA1c (*primary outcome*) at 6 months, by at least 0.17%. We also hypothesize that a healthy low-carbohydrate dietary pattern will lead to larger 6-month improvements in the following *secondary outcomes*: fasting glucose, systolic blood pressure (BP), total-to-HDL-cholesterol ratio, and body weight. We will also study the effect of the intervention on the 6-month change in the following *exploratory outcomes*: fasting insulin and homeostasis model assessment of insulin resistance (HOMA-IR), diastolic BP, waist circumference, and 10-year estimated CVD risk.

### Trial design {8}

We describe the methods of a 6-month parallel-group randomized controlled trial (1:1 allocation to intervention and control groups). This is a superiority trial, with equal allocation to intervention and control groups.

## Methods: participants, interventions, and outcomes

### Study setting {9}

This study is based out of the Office of Health Research clinic at the Tulane University School of Public Health and Tropical Medicine in New Orleans, LA.

### Eligibility criteria {10}

Trial eligibility criteria (Table 1) have been designed to yield adults with or at high risk of T2DM who are able to safely undertake the intervention and likely to be cooperative with trial requirements. As glucose-lowering medications may interfere with the ability to examine the effect of the dietary intervention on HbA1c and other glycemic outcomes, we excluded individuals who have taken such medications in the past 3 months. We chose to focus on adults with HbA1c of 6.0–6.9%, as these individuals may be more likely than individuals with lower HbA1c to have glycemic changes in response to the intervention, but are still within an HbA1c range in which a reasonable proportion of individuals are not taking diabetes medications.

**Table 1:** Trial eligibility criteria

**Inclusion criteria**	
• Men or women ages 40 to 70 years	
• Any race or ethnicity	
• HbA1c 6.0–6.9%	
• Willing and able to provide informed consent	
**Exclusion criteria**	
• Diagnosed type 1 diabetes mellitus based on patient self-report	
• Use of agents affecting glycemic control (medications for diabetes, oral glucocorticoids) within the past 3 months prior to enrolment based on patient self-report	
• Medical condition in which low-carbohydrate diet may not be advised (estimated glomerular filtration rate (eGFR) ≤ 45 mL/min/1.73 m^2^, which is close to the 5th percentile of eGFR among non-diseased individuals of 70 years of age [36], self-report of liver disease due to hepatitis or alcohol; osteoporosis; untreated thyroid disease; gout; or cancer (other than non-melanoma skin cancer) requiring treatment in the past year, unless prognosis is excellent)	
• Factors that may affect HbA1c: hemoglobin < 11 mg/dL (cutpoint for moderate-to-severe anemia, which could lead to falsely elevated or lowered HbA1c) [37], recent blood donation or blood transfusion (self-report, past 4 months), human immunodeficiency virus (self-report) [38]	
• Self-reported history of intensive care unit stay due to coronavirus disease 2019 (COVID-19) in the past 3 months, as severe COVID-19 may affect blood glucose levels	
• Allergies to nuts	
• For women, current pregnancy, breastfeeding, or plans to become pregnant during the study	
• Consumption of ≥ 21 alcoholic drinks per week or consumption of ≥ 6 drinks per occasion	
• For continuous glucose monitor (CGM) collection at the end of the study period only: known allergy to adhesives or other products involved with CGM use (e.g., skin disinfectants), current pregnancy, currently on hemodialysis or peritoneal dialysis, or people with other implanted medical devices (e.g., a pacemaker)	
• Current or planned residence making it difficult to meet trial requirements (due to distance from study site and/or challenges regularly traveling to site)	
• Current participation in lifestyle or pharmaceutical trial	
• Participation of another household member in the study; employees or persons living with employees of the study	
• Other concerns regarding ability to meet trial requirements, at the discretion of the principal investigator or study coordinator	

### Who will take informed consent? {26a}

At screening visit, study staff will conduct informed consent; participants will sign consent forms.

### Additional consent provisions for collection and use of participant data and biological specimens {26b}

Consent includes options to give permission: (1) for blood samples to be stored for future studies, (2) to be contacted for future studies, (3) for genetic testing of biological specimens, (4) to allow de-identified information to be shared for genome-wide association studies, (5) for collection of stool specimens for measurement of gut microbial changes, and (6) to wear a continuous glucose monitor.

## Interventions

### Explanation for the choice of comparators {6b}

Rationale for comparators is described in the “Background and rationale {6a}” section.

### Intervention description {11a}

#### Low-carbohydrate dietary intervention group

Participants randomized to the low-carbohydrate intervention group will receive behavioral counseling and key supplemental food. Phase I (*Go Low*) will occur for the first 3 months (target < 40 g digestible carbohydrates per day). Key recommended foods include non-starchy vegetables (e.g., broccoli, spinach, brussels sprouts, cauliflower), fish, poultry, lean meat, eggs, olive oil and other plant-based unsaturated oils, unsweetened/unsalted nuts and seeds, nut butters, avocados, and moderate consumption of cheese, unsweetened Greek yogurt, and low-carbohydrate milk. We will recommend limiting or avoiding other dairy, fruits, legumes, beans, and grains. During phase II (*Keep it Low*; months 4 through 6), the net carbohydrate goal is < 40 to < 60 g (participants instructed to choose lowest feasible target). With the multi-phased intervention, participants can see how they feel on the more restrictive diet and, if they choose to increase intake, see how they respond to small increases in carbohydrate consumption [39].

Participants will receive a handbook with diet guidelines and recipes and will prepare meals based on guidelines. Materials are modified from published research [40]. The interventionist will instruct participants to reduce digestible carbohydrate intake by increasing protein and unsaturated fat consumption. The interventionist will focus on setting specific, measurable, achievable, relevant, and time-specific (SMART) goals with each participant. At baseline, participants will receive the following: one 1-L container of olive oil, 3 cans of green beans, 3 cans of tomatoes, and samples of a non-sugar sweetener. Throughout the intervention, participants will also receive 4 oz of walnuts, 1 oz of almonds, 2 low-carbohydrate bars, 2 low-carbohydrate shakes, and 1 can of tuna per week and additional samples of non-sugar sweetener.

The *Go Low* phase involves weekly individual counseling sessions for the first 4 weeks, followed by four group sessions held every other week with phone follow-ups occurring in between group sessions (four phone follow-ups). During the *Keep it Low* phase, participants will attend three monthly group sessions and have three telephone follow-ups.

#### Usual diet group

At randomization, participants in the usual diet group will receive written information with standard dietary advice; they will not receive ongoing dietary recommendations during the study. To encourage participation, participants in this group will be offered optional monthly educational sessions on topics unrelated to the intervention.

#### Physical activity

At baseline, both groups will receive written information on standard physical activity recommendations.

### Criteria for discontinuing or modifying allocated interventions {11b}

Participants with HbA1c > 7.9% will be referred to a clinician for follow-up [41]; we will continue to intervene on and collect data from these participants.

### Strategies to improve adherence to interventions {11c}

#### Strategies to improve adherence to the intervention

At screening, staff will conduct a compliance questionnaire. Potential participants unwilling or unable to make necessary dietary changes will not be enrolled. Participants will be provided with recipes and with supplemental food for the intervention diet. The intervention is process-oriented and helps participants develop realistic goals and a reasonable plan that involves making small yet effective changes.

#### Strategies to monitor adherence to the intervention

Adherence to intervention sessions will be measured by recording the number of sessions participants attend. Adherence to dietary recommendations will be assessed using two 24-h dietary recalls from all participants at baseline, 3 months, and 6 months (six total recalls). For each time point, one recall will reflect weekday and other weekend day consumption; recalls will be at least 2 days apart. We will use computer software (Nutrition Data System for Research [NDSR], Nutrition Coordinating Center (NCC), University of Minnesota) [42]. Adherence to dietary recommendations will also be assessed by measuring ketones in spot urine collected at randomization, 3-month, and 6-month visits.

### Relevant concomitant care permitted or prohibited during the trial {11d}

Participants will continue to receive care from their usual healthcare providers.

### Provisions for post-trial care {30}

This study does not provide post-trial care.

### Outcomes {12}

The *primary outcome* will be the difference in HbA1c change from baseline to 6 months between the intervention and usual diet groups. *Secondary outcomes* are the difference in the change of fasting glucose, systolic BP, total-to-HDL-cholesterol ratio, and body weight between the intervention and usual diet groups from baseline to 6 months. *Exploratory outcomes* are the difference in the change of fasting insulin and HOMA-IR, diastolic BP, waist circumference, and estimated 10-year CVD risk [43] between the intervention and usual diet groups from baseline to 6 months. Based on power calculations that assume 95% follow-up (96% follow-up to-date), we estimate 80% power to detect a difference in change of HbA1c of 0.17%. In a follow-up analysis of the DPP, a 0.17% decrement in HbA1c at 6 months was associated with a 17% lower risk of incident T2DM [44].

### Participant timeline {13}

**Fig. 1:**
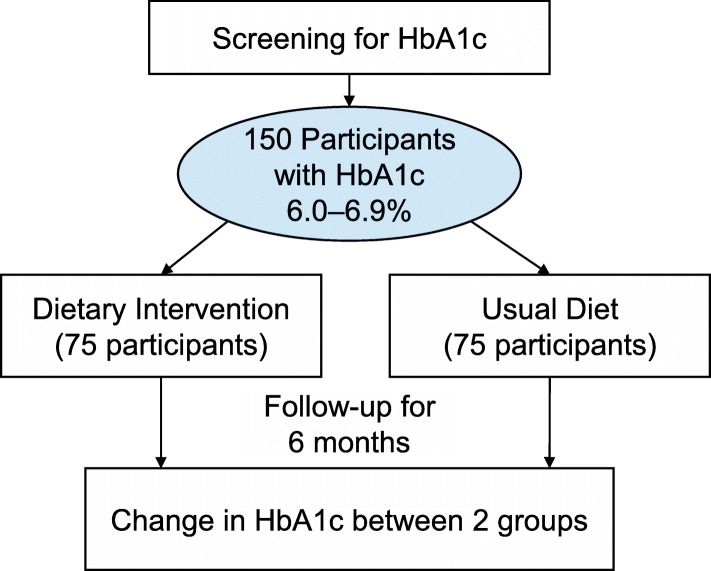
Study design

Table 2 shows the schedule of pre-screening, screening, enrolment/randomization, interventions, and data collection for study participants. Figure 1 shows the overall timeline of the study.

**Table 2:**
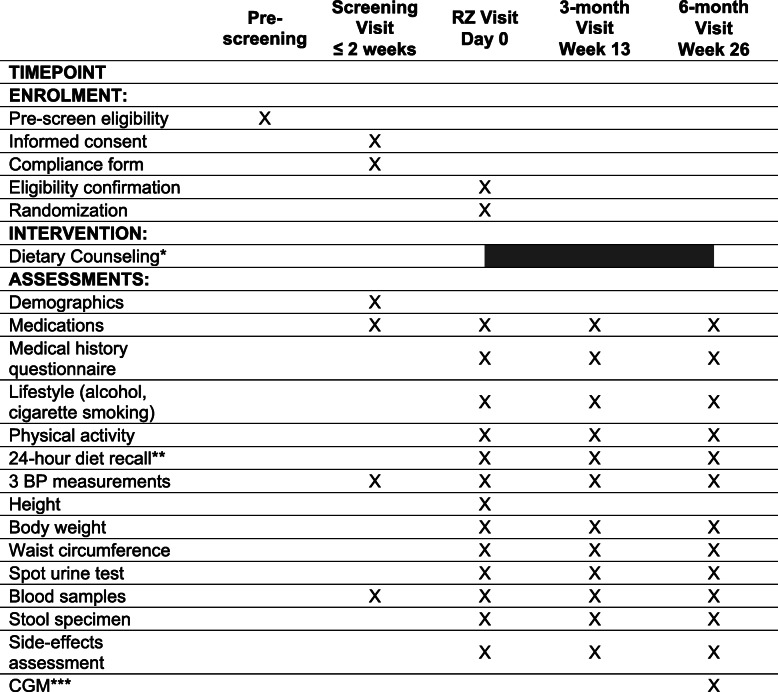
Participant timeline: enrolment, interventions, and assessments

Randomization visit is at least 2 days after screening visit

*RZ* randomization, *BP* blood pressure, *CGM* continuous glucose monitor

*Dietary counseling starts within 2 weeks of randomization and is only provided to the low-carbohydrate dietary intervention arm

**Two 24-h dietary recalls will be collected; one covering a weekend and the other a weekday

***The CGM will be worn for up to 14 days prior to the 6-month visit and returned at the visit or by mail. Participants will come in for a 6-month CGM visit (prior to the 6-month visit) to have the CGM inserted

### Sample size {14}

The primary outcome will be the difference in the change of HbA1c between the intervention and usual diet groups. Pilot data from our group suggests that the standard deviation (SD) of 6-month change in HbA1c is 0.35%, with 6-month follow-up > 95% (follow-up rate to-date is 96%). Based on an estimated standard deviation of change in HbA1c of 0.35% and 95% follow-up, we would need a total of 150 participants (142 participants with follow-up data) to have 80% power to detect a difference in the change in HbA1c from baseline to 6 months of 0.17% between the intervention and usual diet groups (unpaired *t* tests, two-sided alpha of 0.05).

### Recruitment {15}

We will recruit study participants from the greater New Orleans area. The primary focus of recruitment is mass mailing to individuals in the greater New Orleans area, which has successfully been used to recruit for research studies in the same geographic area [40, 45, 46]. Interested individuals will be pre-screened, and those at elevated risk for prediabetes or T2DM (based on American Diabetes Association risk score) will be invited for screening [47]. A secondary recruitment approach involves recruiting outpatients from local hospitals via electronic medical record search.

## Assignment of interventions: allocation

### Sequence generation {16a}

Eligible participants will be randomized to the intervention or control group in a 1:1 allocation ratio (randomized sequence generated by computer program prior to start of the study). Randomization will be stratified by sex, using a random block group size (group size of 4 or 6).

### Concealment mechanism {16b}

The randomization list will be only accessible to staff in the methodology/biostatistics unit who are not physically located at the clinic and are not directly involved in the study. After ascertaining eligibility, clinic staff will obtain randomization assignment from the methodology/biostatistics unit (randomization either obtained by opening a sealed envelope from the methodology/biostatistics unit or by phoning the methodology/biostatistics unit at the time of randomization).

### Implementation {16c}

A statistician not otherwise involved in the study will generate allocation sequence. Once eligibility has been confirmed, study staff will assign participants to the intervention or control group.

## Assignment of interventions: blinding

### Who will be blinded {17a}

Laboratory analysts measuring the primary outcome (HbA1c) and data analysts will be blinded to assignment of participants.

### Procedure for unblinding if needed {17b}

Emergency unblinding will not be required.

## Data collection and management

### Plans for assessment and collection of outcomes {18a}

#### Study visits

After the initial pre-screening interview, in which data on medications and medical conditions will be collected, there will be one screening visit, one randomization visit, a follow-up study visit at 3 months, and a termination visit at 6 months. Table 2 summarizes study procedures and data collected at each visit.

#### Data collection

##### Questionnaires

The following questionnaires will be collected: demographics, medical history, symptom, medication tracking, health behavior (smoking and alcohol consumption), and the International Physical Activity Questionnaire Short Form [48].

##### BP and anthropometric measures

BP will be measured 3 times at screening visit and each follow-up visit. BP will be measured by standard automated blood pressure measurement device (OMRON HEM-907 XL) after participants rest quietly for ≥ 5 min; the 3 BP measurements will be averaged. Height will be measured by wall-mounted stadiometer (Quick Medical; nearest 0.5 cm; first measurement recorded), weight by calibrated digital Detecto Bariatric scale (nearest 0.1 kg; participants wearing light clothing; first measurement recorded), and waist circumference (post-expiratory; 1 cm above the top of the navel) with a Gulick anthropometric tape measure (nearest 0.1 cm; first measurement recorded).

##### Blood samples

At screening visit, non-fasting blood samples will be collected. Fasting blood samples will be collected at randomization, 3-month, and 6-month visits. Fasting blood samples will be stored at − 80 °C until measurement following trial completion. HbA1c will be measured at LabCorp (turbidimetric inhibition immunoassay; Roche Tina Quant; fresh samples) from whole blood collected at screening, 3-month, and 6-month visits. Following trial completion, to improve precision of HbA1c estimates, HbA1c will also be measured at the Diabetes Diagnostic Laboratory (ion-exchange method; Tosoh G8 HPLC Analyzer; frozen samples) from whole blood collected at randomization, 3-month, and 6-month visits. Samples with Hb variants (C, D, E, S) that are measured at the Diabetes Diagnostic Laboratory will be reflexed for measurement by boronate affinity HPLC (Trinity Premier Hb9210) [49].

All other markers described below will be measured following trial completion at the Tulane Office of Health Research Clinical Laboratory. Plasma glucose will be measured by hexokinase method, fasting plasma insulin by ELISA, and serum total cholesterol and triglycerides by enzymatic procedures. Serum HDL-cholesterol will be measured by a combined procedure of heparin-calcium precipitation of apo-B containing lipoproteins and agarose gel electrophoresis of lipoproteins [50]. HOMA-IR will be calculated as fasting insulin (μIU/mL) × fasting glucose (mmol/L)/22.5. The coefficients of variation on blind duplicate samples for the methods used are < 2% for HbA1c, < 3% for total cholesterol, < 5% for HDL-cholesterol, < 3% for glucose, and < 10% (intra-assay) and < 12% (inter-assay) for insulin. For HbA1c, CV is < 3% for LabCorp. At the Diabetes Diagnostic Laboratory, CV for HbA1c from quality control samples is < 1% for short-term (3-month period) and < 2.5% for long-term (years). Prior research has reported HbA1c in frozen whole blood is stable for as long as 10 years [51, 52]. Ten percent blind, duplicate samples will be selected prior to blood drawing.

##### Dietary measurements and spot urine collection

Dietary recalls and spot urine specimens will be collected as described in the “Strategies to monitor adherence to the intervention” section.

##### Stool specimens

Stool samples will be collected with a stool collection kit (OMNIgene GUT) following Human Microbiome Project protocol [53, 54]. Stool samples will be stored at − 80 °C.

##### Continuous glucose monitors (CGM) substudy

Individuals willing to wear a CGM will come to the clinic 2 weeks before the 6-month visit for insertion and activation of the Abbott FreeStyle Libre Pro [55]. For calculation of coefficient of variation, a subset of participants who wear the CGM will be asked to wear two CGMs at the same time, one on each arm.

### Plans to promote participant retention and complete follow-up {18b}

Study visits will be scheduled at participants’ convenience and to minimize visit waiting times and trips; reminders will be made prior to visits; free parking, modest incentives, and gifts will be given to participants; enthusiastic staff with excellent interpersonal skills will be hired. The “Intervention description {11a}” section describes additional approaches to promote retention.

### Data management {19}

Personnel will double-enter study data using a secure REDCap database [56, 57]. Discrepant entries will be checked against paper forms.

### Confidentiality {27}

Confidentiality of study data will be maintained via unique, encrypted study identification numbers in the study database. Study forms will be kept in locked rooms, accessible only by study staff.

### Plans for collection, laboratory evaluation, and storage of biological specimens for genetic or molecular analysis in this trial/future use {33}

Fasting blood samples and stool samples will be stored at − 80 °C for future potential analyses, such as metabolomics, genetic, or microbiome analyses.

## Statistical methods

### Statistical methods for primary and secondary outcomes {20a}

We will compare means, medians, and percentages of baseline variables across the intervention and usual diet groups to assess similarity of these groups at randomization.

The data will be analyzed using intention to treat. We will test primary study hypothesis that there is a greater reduction in HbA1c from baseline to 6 months in the intervention group than in the usual diet group. To do this, we will use linear regression with change in HbA1c from baseline to 6 months as the dependent variable. We will use a similar approach to test the difference in the change between baseline and 6-month values of secondary and exploratory outcomes. For each outcome, we will test modeling assumptions and consider transformation of outcomes to meet modeling assumptions.

We will use Bland-Altman plots to explore whether there is a systematic difference in HbA1c measured by LabCorp compared with HbA1c measured by the Diabetes Diagnostic Lab. If there is not a systematic difference in HbA1c measured at these two laboratories, to improve precision in the estimate of the primary outcome (difference in change in HbA1c between the intervention and control groups), we will use the weighted average of change in HbA1c measures from the two labs as the outcome (weights defined as the ratio of the variance for each lab over the sum of the variance) [58].

### Interim analyses {21b}

There will not be interim analyses.

### Methods for additional analyses {20b}

In exploratory analyses, we will use the same modeling approach to test difference in the change between baseline and 3-month values of outcomes in the intervention compared with control groups. We will explore use of mixed effects models to assess effect of the intervention on 6-month net change in outcomes.

### Methods in analysis to handle protocol non-adherence and any statistical methods to handle missing data {20c}

We will perform sensitivity analyses using multiple imputation by Markov-chain Monte Carlo techniques to impute missing values of outcomes (using covariates; assuming arbitrary missing pattern and multivariate normal distribution for the data) [59]. We will compare point estimates and confidence intervals from this analysis with our primary findings from complete case analysis.

### Plans to give access to the full protocol, participant-level data, and statistical code {31c}

The full protocol and statistical code will be available upon request.

## Oversight and monitoring

### Composition of the coordinating center and trial steering committee {5d}

Study organization consists of the study steering committee (KSD, LAB, LQ, JH), clinical center, biostatistics/methodology unit, and laboratory. The steering committee will finalize the protocol and will regularly communicate via telephone, email, and meetings. The biostatistics/methodology unit will generate randomized allocation and provide guidance on statistical analyses. Weekly investigator and clinic staff meetings will be held.

### Composition of the data monitoring committee, its role and reporting structure {21a}

A Data and Safety Monitoring Board (DSMB), which consists of three experts who are not otherwise affiliated with the study, will meet twice per year and review reports that include data on recruitment, randomization, and adherence. The DSMB makes recommendations concerning the continuation, modification, or termination of the trial.

### Adverse event reporting and harms {22}

At randomization, 3-month, and 6-month visits, participants will complete symptom questionnaires to identify complaints and will report hospitalizations at follow-up visits. Study staff will collect data on spontaneously reported adverse events. Severe adverse events will be promptly reported to the DSMB. Management of adverse effects will be based on participant protection and safety. If severe adverse effects are reported, carbohydrate intake will be examined and, if appropriate, altered to mitigate the adverse effect. In extreme cases, participants will be instructed to stop the intervention.

### Frequency and plans for auditing trial conduct {23}

Throughout the trial, the study may be audited by the Tulane University Institutional Review Board (IRB).

### Plans for communicating important protocol amendments to relevant parties (e.g., trial participants, ethical committees) {25}

Protocol amendments will be approved of by the IRB and DSMB prior to implementation. If relevant, current participants will be informed of protocol modifications. The ClinicalTrials.gov registry for this study will be updated with important protocol amendments.

## Dissemination plans {31a}

The results of this study will be disseminated through publication of the findings at scientific meetings and in peer-reviewed publications.

## Discussion

Lifestyle modifications play an important role in T2DM prevention [12], with a standard dietary approach focused on reducing caloric and total fat intake. However, types of fat may be more important than quantity of fat [20, 21]. Additionally, low-to-moderate carbohydrate diets are effective at reducing HbA1c among individuals with T2DM [26]. However, research focused on effects of low-carbohydrate diets on glycemic outcomes among individuals with prediabetes is limited to pilot or non-randomized studies [32–35].

As low-to-moderate carbohydrate diets lead to weight loss in general populations [22, 23] and improve glycemic control among people with T2DM [26], it is a high public health priority to study glycemic effects of such diets in people with elevated HbA1c who are not currently on glucose-lowering medications. This trial will study the effect of a healthy low-carbohydrate diet through behavioral intervention and key food supplementation among individuals not taking glucose-lowering medications who have elevated HbA1c (HbA1c 6.0–6.9%). This low-carbohydrate diet is characterized by abundant unsaturated fats and protein, high-fiber foods such as non-starchy vegetables and nuts, and minimal refined carbohydrates.

We expect the results from this study have the potential to lead to new horizons for developing and implementing dietary approaches (other than the most frequently used reduced fat diet) that will substantially reduce risk of cardiometabolic disease among adults with or at high risk of T2D. The proposed intervention promotes consumption of dietary components thought to reduce risk of cardiometabolic disease, and the behavior change intervention has expected applicability in clinical practice. Findings may provide support for promoting alternative dietary styles among individuals with or at high risk of T2DM and could potentially be used to inform alternate dietary interventions in diabetes prevention programs.

### Trial status

Protocol version number and date: V2.2 September 14, 2020

Recruitment start date: September 25, 2018

Recruitment end date (anticipated): January 1, 2021

To-date, 150 of 150 anticipated participants have been enrolled into the study.

## Abbreviations

BP: Blood pressure
CVD: Cardiovascular disease
CDC: Centers for Disease Control
CGM: Continuous glucose monitor
DSMB: Data and Safety Monitoring Board
DPP: Diabetes Prevention Program
HbA1c: Hemoglobin A1c
HDL: High density lipoprotein
HOMA-IR: Homeostasis model assessment of insulin resistance
IFG: Impaired fasting glucose
IGT: Impaired glucose tolerance
IRB: Institutional Review Board
NDSR: Nutrition Data System for Research
NCC: Nutrition Coordinating Center
SMART: Specific, measurable, achievable, relevant, and time-specific
T2DM: Type 2 diabetes mellitus
